# Caretakers' perspectives of paediatric TB and implications for care-seeking behaviours in Southern Mozambique

**DOI:** 10.1371/journal.pone.0182213

**Published:** 2017-09-14

**Authors:** Carolina Mindu, Elisa López-Varela, Yara Alonso-Menendez, Yolanda Mausse, Orvalho Joaquim Augusto, Kizito Gondo, Jose Múñoz, Jahit Sacarlal, Alberto L. García-Basteiro, Pedro L. Alonso, Khátia Munguambe

**Affiliations:** 1 Centro de Investigação em Saúde de Manhiça (CISM), Cambeve, Maputo, Mozambique; 2 ISGlobal, Barcelona Global Health Institute, Hospital Clınic – Universitat de Barcelona, Barcelona, Spain; 3 Faculdade de Medicina, Universidade Eduardo Mondlane (UEM), Maputo, Mozambique; 4 Amsterdam Institute for Global Health and Development (AIGHD), Academic Medical Center, Amsterdam, The Netherlands; Centre for International Child Health, University Department of Paediatrics, Royal Children's Hospital, Melbourne, Australia, AUSTRALIA

## Abstract

**Background:**

Tuberculosis (TB) remains an important public health concern, especially in poorly resourced settings. TB diagnosis is challenging, particularly for children, who are the most vulnerable to its′ impacts. Lack of knowledge and awareness of the disease compromises prompt diagnosis and treatment compliance.

**Objective:**

To gain insights regarding caretakers′ knowledge of the aetiology and prevention of paediatric TB in southern Mozambique, to describe their care-seeking behaviours and to assess the acceptability of diagnostic procedures.

**Methods:**

A total of 35 caretakers were interviewed, all of which had children with TB compatible symptoms. Eleven were caretakers of children diagnosed with TB at the health facility, 11 of children for whom TB was excluded as a diagnosis at the health facility and 13 of children with TB compatible symptoms identified in the community. The first two groups took part in a TB incidence study, while the third group did not. All underwent the same semi-structured interviews, the results of which were analysed and compared using content analysis.

**Results:**

Even when confronted with signs suggestive of TB, most caretakers never suspected it or misinterpreted the signs, even among caretakers with TB and TB contacts. There was limited knowledge of TB, except among those undergoing treatment. The transgression of social norms was often presented as an explanation for TB in parallel to medically sound causes. The use of traditional care for prevention is widespread, but it varied for treatment purposes. TB diagnostic procedures were considered painful but were unanimously tolerated.

**Conclusions and significance:**

Misconceptions of paediatric TB, associated complex care-seeking itineraries and negative feelings of the diagnostic procedures may result in delays, low adherence and lost to follow-up, which needs to be addressed by adequately framed health promotion approaches.

## Introduction

Tuberculosis (TB) is an under-recognized but important cause of morbidity and mortality among children from high TB burden countries[1,2]. Estimates indicate that the National TB Control Programs (NTP) only capture one-third of paediatric TB cases in these settings [3,4]. This is mostly due to the difficulties in diagnosing paediatric TB, in turn leading to underdiagnosis, misdiagnosis or delayed diagnosis. Not only it is a challenge to obtain samples from young children for microbiological examination but also traditional microbiologic tests perform poorly. Additionally, the spectrum of TB disease in children is broad, with a significant confounding effect of HIV on the clinical presentation[5], and an overlap with other common conditions such as pneumonia and severe malnutrition. Because TB commonly affects children in resource-limited settings, difficulty in reaching an accurate TB diagnosis is aggravated by the complex patterns of care seeking[6,7], increasing the risk of rapid disease progression and mortality in young children[5].

Few studies have examined the perceptions of local communities regarding the aetiology and prevention of TB[8–10], and even fewer address paediatric TB[11,12]. Understanding how TB is conceptualised is crucial, not only to comprehend the causes of treatment delays but also to inform strategies to increase awareness and change behaviour. Although most of the available qualitative research on TB has focused on the behavioural perspective, it has mainly been directed at improving adherence to health facility based treatment among adults[13–16]. In many settings, adult TB patients have reported some form of self-treatment or treatment prescribed by traditional healers before presentation to the formal health services[15,17,18]. There is still little understanding of the care-seeking practices particular to paediatric TB as well as the patterns that these itineraries follow. Understanding caregivers' recognition of paediatric TB could thus contribute to improved detection and treatment of these cases.

This study sought to describe local understandings of the aetiology and prevention of paediatric TB among caretakers in Southern Mozambique, identify the care-seeking practices related to this disease and explore the relationship between the former and the latter. Further, it aimed to evaluate the acceptability of paediatric TB diagnostic procedures among caregivers who seek care at health facilities.

## Materials and methods

### Study site and population

The study was conducted in southern Mozambique, in f Manhiça and Bilene-Macia districts. Mozambique has one of the highest TB and HIV burdens worldwide[19]. According to the latest World Health Organization (WHO) Global TB report, it ranks third and fourth in TB mortality among and HIV-positive population and overall TB incidence respectively[20]. Most new-born children in the country receive Bacille Calmette-Guerin (BCG) vaccination, which has a coverage of around 86–94%[21,22]. TB treatment, including paediatric combined formulations, is offered free of charge by the NTP.

Manhiça is a rural District within Maputo Province, where the Centro de Investigação em Saúde de Manhiça (CISM) runs a Health and Demographic Surveillance System (HDSS) of a population served by the Manhiça District Hospital (MDH) and 10 peripheral health centres [23]. At the time of the study, the HDSS covered a population of around 92,000 inhabitants (out of a total population of 160,000), of which approximately 11% were <3yr. The population belongs mainly to the Changana ethnic group. Community-based studies have shown an HIV prevalence of 39.9% among individuals aged 18–47 years[24] and a minimum community-based TB incidence rate of 470 per 100.000 person-year under the age of three[25].

The second study site, the neighbouring district of Bilene-Macia located in Gaza Province, is also a rural area belonging to the Changana ethnic group. It has an area of 2.180 Km^2^ and 151,548 inhabitants[26]. The reasons for extending the study area to Bilene-Macia are the following. Firstly, given that it has an ethnic background similar to Manhiça, it allows for comparison at a cultural level. Secondly, it differs from Manhiça in that it does not have a history of clinical research. Thirdly, the district of Bilene-Macia has a lower formal health service coverage than Manhiça, which suggests possible differences regarding care-seeking practices.

### Study design and procedures

This was a qualitative study which recruited; (i) caretakers of children with presumptive TB, in whom TB was ruled out after clinical evaluation at the MDH (Group A); (ii) caretakers of children diagnosed with TB at the MDH (Group B); and (iii) caretakers of children with TB compatible symptoms identified by traditional healers (Group C). A distinction between groups A and B was made in order to evaluate the potential influence of being under treatment, implying regular contact with the NTP which includes some counselling (group B), on knowledge of TB compared to not being under treatment as a result of ruling out TB (group A).

Groups A and B were recruited and interviewed in Manhiça in parallel to a larger prospective childhood TB incidence study[27], which used active and passive case detection strategies to identify cases with TB compatible symptoms (Box 1). Presumptive cases were then evaluated through physical examination, tuberculin skin and HIV testing, chest X-Ray as well as microbiological evaluation of samples obtained through same day induced sputum (IS) with nasopharyngeal suction and gastric aspirate (GA). A sub-sample of these cases was selected to take part in the qualitative study. Recruitment for the qualitative study was done by requesting the clinician for caretakers ≥ 18 years old, accompanying children between 1–3 years with criteria to participate in the incidence study, residing in the district of Manhiça and willing to take part in the qualitative study. Assignment to group A or B occurred after being examined for TB and either having the disease ruled out (group A) or receiving a positive TB diagnosis (group B).

**Box 1. Signs and symptoms considered TB compatible for the purpose of this study**

Cough for ≥14 days not responding to a course of antibiotics

Referred fever for ≥14 days after common causes like malaria were excluded

Weight loss/failure to thrive (defined as under 60% weight for height, failure to gain weight for >2 months, any loss of weight unresponsive to nutritional rehabilitation)

Unexplained wheeze ≥14 days not responding to standard treatments

Lower respiratory tract infection ≥14 days not responding to antibiotics after 72 hours

TB exposure in the last 12 months

Symptoms compatible with EPTB (such as painless enlarged lymph nodes with or without fistula formation ≥14 days, arthritis, gibbus, meningitis, effusion or unexplained hematuria, dysuria or polaquiuria for ≥21 days)

A third group (group C) consisting of caretakers of children between 1–3 years with TB compatible symptoms recruited outside the study area of the TB incidence study was included in order to explore the same issues but outside the framework of a clinical research project. For this purpose, a group of traditional healers in the district of Bilene-Macia was trained to screen presumptive TB patients. The training enabled traditional healers to identify and refer caretakers with children presenting a combination of signs, symptoms and other precedents compatible TB (Box 1). Besides identification of caretakers during their visits to the TH, study fieldworkers, in collaboration with THs and district health authorities, actively sought cases within the TH′s catchment areas.

A minimum sample size of 10 caretakers per group was foreseen to fulfill the study objectives. The sample size was based on previous experience in reaching saturation while conducting similarly designed qualitative studies coupled by indications from the literature [6, 7, 11].

### Data collection and analysis

Data was collected through semi-structured interviews (SSI) by two trained Mozambican social science research assistants (CM and YM), supported by two local interviewers.

Caretakers were invited for a SSI focused on their knowledge of TB (aetiology and prevention), care seeking practices (including the extent to which they sought alternative care), initial interpretation of their child's symptoms, itinerary and experiences from perceived onset of the illness until the moment of diagnosis, and their assessment of the diagnostic procedure and results (see Box 2 for overview of questions). Participants were visited at their households, explained the nature of the study, invited to participate and consulted on the suitable date to conduct the interview. The majority of interviews took place at the participants' homes. Choice of language, which was mainly XiChangana, was determined by participants’ convenience. All interviews were tape-recorded; recordings were transcribed *verbatim*, and translated simultaneously from Changana to Portuguese by the interviewers themselves. Quality control was ensured by a review of 40% of the transcripts against the original audio recordings to confirm accuracy. Content analysis was performed by synthesising findings according to pre-determined themes and comparisons across the three groups.

**Box 2. Interview guiding questions**

How do you think TB is transmitted?

What do you believe causes it?

Is it possible to prevent it? How so?

What made you take the child to the hospital?

What symptoms did s/he present?

How did you interpret these? Did you suspect of any illness?

How did you feel about the diagnostic procedure?

Did you seek any other treatment before the hospital? If so, which kind?

What is your preferred form of treatment?

### Ethical considerations

The study protocol was approved by the CISM Institutional Review Board (IRB ref: CIBS_CISM/02/12) and the Ethics Review Committee of the Hospital Clinic of Barcelona. Participation was voluntary and written informed consent was obtained from all the caretakers participating in the study. All children with warning signs for TB and other illnesses found in the community were actively referred to the nearest health facility.

## Results

### Characteristics of respondents

A total of 35 participants were included in the study (11 in groups A and B, respectively, and 13 in group C). All caregivers were female, and 90% of them were the children′s mothers. Table 1 presents the socio-demographic characteristics of the caregivers and their children.

**Table 1:** Socio-demographic characteristics of caregivers and children.

Characteristics of Caregiver^2^	n (%)
Relationship	
Parent (mother)	28 (90)
Aunt	2 (6)
Grandmother	1 (3)
Formal education	
Secondary	18 (58)
Primary	9 (29)
None	2 (6)
Unknown	2 (6)
Occupation	
Formally unemployed	11 (35)
Farmer	6 (19)
Unknown	6 (19)
Formally employed	2 (6)
Sugar estate worker	1 (3)
Student	1 (3)
Seller	1 (3)
Marital status	
Married	11 (35)
Unknown	16 (52)
Divorced	2 (6)
Single	2 (6)
**Characteristics of Child**	
Sex	
Male	18 (51)
Female	17 (49)
**Characteristics of Household**^3^	
N° people	
<5	4 (22)
5–10	10 (56)
>10	4 (22)
N° children	
1–5	12 (67)
>5	6 (33)
TB contact	7 (32)
Caretaker	4
Household contact	3

Since the majority of participants of the incidence study were recruited passively (only 6% were recruited actively), all participants of group A and B, with the exception of one, were recruited from this group.

^2^ We were unable to identify demographic data of 4 of the caregivers from groups A and B and thus percentages are calculated over a denominator of N = 31.

^3^ This data was only collected for those that participated in the incidence study (groups A and B)

### Identification and interpretation of TB compatible signs and symptoms

When asked about signs and symptoms presented by the child and reported triggers of care-seeking, the most common complaints were fever, prolonged cough, diarrhoea, respiratory distress and oedema. The most cited suspected illness was asthma, also referred as 'illness of the chest' (*xifuva*) (n = 13), followed by HIV (n = 5), 'lack of blood' (*kuhela ngati*) (n = 4), flu (n = 4), malaria (n = 2) and 'illness of the moon' (*mavabji ya n'weti*)(n = 2). The latter is essentially characterised by convulsions, therefore often translated as epilepsy.

“*Eh, I thought of all illnesses, even HIV. This is why I had to go test him and they said he didn't have it [HIV]*”

– Caretaker, Group B

Only two caretakers suspected TB (one of which was a TB patient herself); the first cited TB on its own and the second cited it along other possibilities, such as HIV and *nkholhole—*a locally identified illness commonly associated with adults, often translated as TB, described as intensive, prolonged coughing (sometimes with blood expectoration), chills and loss of appetite. On the other hand, the rest of the caretakers who were either TB patients themselves or had household TB contacts did not suspect of TB (n = 6).

Approximately a third of the caretakers stated they suspected no illness (n = 10). Several of them cited reasons for this, including that the symptoms were not worrisome enough, that it was simply expected that children became ill, or that there was no apparent cause (for example, a death in the household or vicinity, or the presence of adultery). (See Table 2)

“*… I didn′t suspect anything […] I wouldn′t think that something like this could have happened because I didn't think that in my house there are problems… The problem is when there is a death, and you fail [to follow the rituals]…it may happen that the child falls [ill].*”

– Caretaker, Group A

**Table 2:** Condition suspected by mothers and caretakers of presumptive TB cases.

Suspected condition (nº of caretakers)	Signs described (n° of caretakers)
None (10)	Low weight/loss of appetite (7), fever (5), cough (6), seizures (0), diarrhoea (2), oedema (2), rhinorrhoea (1), headache (1), epistaxis (1), difficulty breathing (1)
HIV (5)	Low weight/loss of appetite (2), cough (3), diarrhoea (4), oedema (1), fever (1), vomiting (1), loss of strength (0), rhinorrhoea (1), difficulty breathing (2), bad general appearance (1)
Asthma (13)	Low weight/loss of appetite (4), cough (13), diarrhoea (2), bad general appearance (2), difficulty breathing (8), fever (4), vomiting (2), chest pain (1)
TB (2)	Low weight/loss of appetite (1), cough (2), diarrhoea (1), oedema (1), rhinorrhoea (0), loss strength (0)
Flu (4)	Cough (4), low weight (1), fever (3), bad general appearance (1), rhinorrhoea (1), difficulties breathing (1), chest pain (1), vomiting (1)
Illness of the Moon (2)	Cough (1), fever (1), difficulty breathing (1), seizures (1), low weight (2)
Lack of blood (4)	Fever (2), oedema (2), difficulty breathing (2), cough (2), vomiting (1), low weight (1), diarrhoea (1)
Malaria (2)	Fever (2), cough (2), difficulties breathing (1)

### Knowledge and perceptions on the aetiology and prevention of TB

When enquired on their knowledge of the aetiology of TB, many respondents gave several and distinct answers. Amongst groups A and B, over half of the caretakers showed some knowledge about the aetiology of TB (n = 12), which was in-line with the common health promotion messages. These groups′ medically sound knowledge on TB aetiology or transmission modes included coughing (n = 4), coughing and sharing food or utensils (n ***=*** 4), or contact with a TB-infected person (n ***=*** 4). However, it is important to note that, except one, all of the caretakers that indicated coughing as a mode of transmission belonged to group B (TB-positive children), and all who suggested contact with a TB-positive person were either TB patients or cohabited with a TB patient.

Of note, many of those who revealed perceptions of TB in line with biomedical knowledge simultaneously provided other radically different answers. A reasonable number of caretakers (n = 8) reported that TB results from the transgression of social norms, such as adultery (n = 3), and failure to comply with rituals (n = 1), including death-related rituals (n = 4). In the latter case, the deceased is a household member, whose death results in vulnerability of family members to TB, often as a consequence of them not performing *kutxinga* (a purification ceremony involving the engagement of a couple of close relatives of the deceased in a sexual act carried out during the mourning period), which, if not performed appropriately, is believed to bring misfortune to the family. In the case of adultery, its presence is perceived to cause the transmission of TB through the sharing of objects.

[Reflecting on her reaction after being informed her son had TB] *"Oh*! *I became surprised 'eh*! *Can you get this illness when you never committed adultery*?*‴*

– Caretaker, Group B

Other mentioned forms of acquiring TB were stepping on infected sputum (n ***=*** 1), through a virus (n = 1), being low weight (n = 1), exposure to dust and cold (n = 1) and the 'separation of lungs' (n = 1). On the other hand, a significant amount of the caretakers (n ***=*** 8) claimed not to know about the aetiology or transmission of TB, despite the fact that three of them did have TB themselves.

Amongst group C, the majority of caretakers (n ***=*** 10) expressed no knowledge of the aetiology of TB, while only one indicated coughing as a mode of transmission. One caretaker stated that a possible cause was sharing food with a mourning person, while another claimed close contact with a person that had committed adultery as a cause of TB.

“*Yes, I thought maybe her blood had finished […]So I said that maybe someone who had committed adultery had given her something, something hot*”

– Caretaker, Group C

Regarding prevention, groups A and B pointed out various possible methods. The most cited form of prevention was avoiding the sharing of food or related utensils (n = 5), followed by avoiding cold temperatures (n = 1), covering the mouth whilst coughing (n = 1), avoiding sleeping beside a TB infected person TB (n = 1), and avoiding sexual intercourse during the lactation period (n = 1). Finally, more than half of caretakers from groups A and B (n = 15) stated they did not know how TB could be prevented.

The majority (n = 11) of caretakers of group C indicated they did not know any possible method of prevention. Only two suggested the consumption of minced garlic as a form of prevention (see Table 3).

**Table 3:** Caretaker′s knowledge and perception on TB aetiology and prevention.

Caregivers (n)	Aetiology (n)	Prevention (n)
Group A—non TB cases (11)*	Unknown (6)	Unknown (9)
	Death (1)	Avoid sharing food (1)
	Dust, cold (1)	Avoid sex during lactation (1)
	Low weight (1)	
	Cough (1)	
	TB contact (1)	
	Virus (1)	
	Separation of the lungs (1)	
Group B—TB cases (11)	Cough, sharing of utensils (4)	Unknown (6)
	Cough (3)	Avoid sharing food/utensils (4)
	TB contact (3)	Avoid sleeping close to TB patient (1)
	Adultery (3)	Having cold drinks (1)
	Death (3)	Cover mouth (1)
	Unknown (2)	
	Stepping on TB infected sputum (1)	
	Ritual neglect (1)	
Group C -non TB cases (13)	Unknown (10)	Unknown (11)
	Cough (1)	
	Death (1)	Consuming minced garlic (2)
	Adultery (1)	

*The numbers of group B at times (in the case of causes and prevention) surpass the total number of caretakers because in a few occasions a given respondent would provide various different answers.

### Care-seeking practices

The majority (n = 18) of caretakers of groups A and B sought alternative care at some point during the child's lifetime. Cultural norms require the performance of the *kutsivelela* ritual on the child soon after birth. This ritual is performed by traditional healers and precedes the administration of a traditional remedy to prevent the aforementioned 'illness of the moon'.

“*He/she may be two months old, one month, and has to take that remedy for… to make the illness [n'weti] disappear… that one of old tradition*”

– Caretaker, Group B

All participants that reported seeking alternative care (N = 18) had administered this remedy to their children. Most (n = 13) reported having sought traditional healers solely for the purpose of prevention and claimed this was the sole remedy their children had ever received from a TH. The remaining reported having sought additional care from traditional healers (n = 3) or administered homemade remedies (n = 2) to treat their child's current illness.

In the case of group C, the majority (n = 9) also reported having sought alternative care through traditional healers at some point during the child's lifetime. Approximately half (n = 4) claimed to have sought traditional healers solely for the purpose of treating their child's current symptoms. The rest claimed it was for prevention (n = 3), or both for prevention and treatment purposes (n = 2). Thus, nearly half (n = 6) of the caretakers of group C had sought traditional healers for treatment purposes, in contrast to merely three caretakers (1/3) in groups A and B.

All caretakers in the three groups had sought care in the formal system at some point. However, those who had also sought alternative care did approach both systems in no particular order; and often in a circular manner. The following is an excerpt from an interview with a caretaker of group C that suitably illustrates this circularity:

Interviewer—When she began to get ill, what was the first thing you did here at home?

*Respondent—I took her to the hospital […] First I gave her conventional medicine […] I saw she was not improving so I gave her traditional medicine and then I saw she was still not responding so I returned to the hospital*.

On perceived availability of care for TB, over half (n = 12) of the caretakers of groups A and B indicated the hospital as the only place where it can be treated. Few (n = 3) had heard that traditional healers could treat it, though they claimed not to believe it. A considerable amount (n = 9) thought that both systems could treat TB. In contrast, only a minority (n = 5) of group C claimed that TB could only be treated at hospitals and the majority (n = 8) believed that both systems could treat TB(20). Caretakers who considered that traditional healers could treat TB did so because they believed that since on the one side there are traditional illnesses and on the other conventional illnesses, each domain has its own sphere of illnesses it can diagnose and treat.

“*You get sick with that illness and you go to the TH, but there are illnesses that do not go according to tradition…*”

– Caretaker, Group B

Regarding treatment preference, all caretakers of groups A and B claimed to prefer hospitals to traditional healers because they considered them financially accessible, safer and more effective. On the other hand, a significant number (n = 4) of caretakers of group C claimed to consider both equally capable and expressed no particular preference. The only concern raised regarding treatment by traditional healers was the financial burden it often supposed, in contrast to public hospitals where TB treatment is free of charge (see Table 4).

**Table 4:** Care-seeking practices.

Practices	Groups A & B (22)	Group C (13)
Sought alternative care	18	9
Prevention only	13	3
Treatment only	0	4
Prevention & Treatment	5	2

### Acceptability of TB diagnosis and diagnostic procedures

Although diagnostic techniques, especially the induced sputum, were perceived by caretakers to be harmful to children, they were in fact tolerated. Table 5 summarises the feelings expressed by mothers and caretakers who witnessed the procedures. Half of the caretakers (n = 11) stated they felt fearful and all felt discomfort while watching what seemed to be a very painful procedure.

“*They did that thing of inserting small tubes and then inserting them in the mouth to take out sputum… Ah! I was scared! I also cried… I was scared because I had never seen that done to a child…*”

– Caretaker, Group A

**Table 5:** Different attitudes and reactions of caretakers facing the invasive TB diagnostic.

	Category	Nr of respondents	Illustrative quotes
Attitude facing procedure	Scared	9	“When they inserted the tubes I got very scared, I did not have the courage to look…”
	Pity/ Worried about child′s reaction	7	“I didn′t look because my heart hurt…the child cried a lot”
	Angry/ sad/ anguished	7	“I was angry… felt sorry for him, because he screamed…I could tell that they were hurting him”
	Neutral	3	“I don′t know, I just wanted to see my daughter get well”
Acceptability	Acceptable	11	“It is acceptable because the child go well after they diagnosed him”
	Acceptable but necessary	15	“It is too invasive and painful, but it is necessary”
	Neutral	1	N/A

Despite their consideration that procedures were invasive, aggressive and painful, all caretakers accepted their need, which was explained in terms of their trust in the formal system and the power of technology.

“*But it is the machines that detect it. Not even the doctor… So it can still happen that I say my child has no illness but when the machine sees it I have to believe it because once he started to take the pills he became better*”

– Caretaker, Group B

In terms of accepting the positive TB result, many (n = 4) caretakers of group B did not believe a child under 3 years could have TB, and thus expressed surprise upon hearing the result. Further reasons for such surprise were the absence of perceived causes, such as close deaths and/or adultery. In contrast, those who already had a close TB contact, or were TB positive themselves, found it easier to accept the results.

## Discussion

This study substantially adds to the yet limited body of evidence regarding paediatric TB from the socio-behavioural perspective[28–32]. Overall, the results of this study indicate very limited awareness of symptoms compatible with paediatric TB amongst caretakers of small children in Southern Mozambique, including those vulnerable or exposed to TB.

It has been well documented that the misinterpretation of symptoms can delay appropriate care seeking [8,10,33]. In the present study, an insignificant number of caretakers across all groups interpreted their child's symptoms as potentially linked to TB, even amongst those who either were TB patients or had a TB contact in the household. However, the most cited illness under suspicion was asthma, followed by HIV, which are both easily, and often, confounded with TB, even by health professionals[30,33]. These results align with a study conducted in Kenya [8]. The confusion with HIV may discourage caretakers from facing the problem, due to the stigma attached to it, resulting in inappropriate care seeking.

A third of the caretakers did not suspect any particular illness. Although many eventually sought care, this process could have potentially resulted in delays. This inability to detect that the child is ill can be further aggravated by the fact that TB symptoms are sub-acute and insidious, being easily perceived as mundane, 'non-alarming' afflictions, which do not justify prompt care-seeking [6,11,34]. Finally, as caretaker's reactions to diagnosis revealed, the recurrent perception of TB as an illness of adults could further contribute to the misinterpretation of symptoms among children.

The results also revealed the lack of knowledge regarding the aetiology of TB; this was especially exacerbated among caretakers of group C. However, despite having participated in a TB screening process, thus having been in contact with TB health professionals, caretakers of group A presented a similarly limited understanding of TB transmission to that of group C. In contrast, caretakers of group B presented a relatively high level of understanding, with over half of respondents reporting coughing as a form of transmission. These results disagree with findings from Asia, according to which only around 40% of caretakers of children recently diagnosed with TB in China and those under treatment of TB in India were aware of the disease transmission modes [11,12]. The differences in knowledge found in Mozambique suggest that group B was more exposed to biomedical knowledge of TB, given that they initiated TB treatment, which implies weekly visits to the NTP accompanied by counselling.

Medically sound knowledge of TB ***was mostly presented*** in parallel to socio-culturally derived knowledge. These alternative accounts of the causes of TB were either related to the presence of a nearby death (either through purification ritual neglect or direct contact with a ***mourning*** person) or the presence of adultery (usually transmitted through the sharing of objects). These two ***accounts*** reflect a perceived connection between the causation of illness and the notion of impurity brought about by a 'heated' act, person or environment. This corroborates with the indigenous theories of contagious diseases documented from various locations, including Southern Mozambique[35], which advance that the lay disease causation models for many bio-medically classified as communicable diseases may also have a quasi-naturalistic component from the lay perspective, rather than the often identified supernatural attributions that include witchcraft and the evil eye [8,33,36]. The association of TB with sexual intercourse in general [33,36] and with adultery in the case of Mozambique is also indicative of a perceived association between HIV and TB.

Regarding the acceptability of the diagnostic procedure, the results reveal a high degree of acceptance that is consistent across groups. The majority of caretakers reported feeling fearful and/or considered the procedure to be invasive, aggressive or painful, yet they all claimed to consider it necessary and thus tolerable. This observation correlates with previous studies assessing other diagnostic/prevention tools undertaken in the same setting[37–40]. These studies concluded that the hospital is highly regarded and that patients often expressed their trust and obedience towards the procedures ('the law of the hospital'). Few studies have aimed to assess the acceptability of TB diagnostic procedures in children, although these findings do correlate with the positive results attained in a study conducted in Peru[41].

Practically all caretakers seek some form of alternative care through TH, although the results revealed important differences between the two sites; one limiting it to prevention and the fulfilment of social norms and the other emphasising treatment. This discrepancy was expected and thus confirms our initial assumptions, which are in turn based on the aforementioned factors that distinguish the two districts; 1) the lower level of formal health-care coverage present in the district of Bilene-Macia, and 2) the history of influence of a research centre on the population of Manhiça. In line with the results of this study in Bilene-Macia, other studies in Sub-Saharan Africa have found a rate of traditional healers use for the treatment for TB to be as high as 40%[17,18].

The results revealed that the care-seeking pattern among those who sought treatment in both the formal and alternative systems consists of a rather circular and complex itinerary, characterized by a continuous exchange between the two. Similar patterns of care seeking have beenfound in studies across Africa[17,34,36,42]. In contrast with the present results, these studies claim that, amongst those seeking alternative care, the itinerary invariably begins with the TH. However, the results of this study indicate that the itinerary may equally begin at the formal or alternative system, revealing three important concerns. Firstly, when the itinerary starts at the TH, this entails delays in reaching the formal system, an issue that is of particular concern for paediatric TB. According to several studies, this delay averages two months [7,34].

Secondly, the stop-over at the traditional healers places pressure on the formal system to successfully treat their patients before losing them, at least temporarily, to the alternative care system. Finally, it implies that those patients seeking both systems of care may be mixing, and most probably alternating between, both treatments, which may further delay or annul the completion of treatment.

These care-seeking patterns are not only relevant in terms of delays in contacting the formal health system but also to treatment adherence and patient loss to follow-up. This study confirms and contextualises results from a previous study on paediatric TB adherence in Manhiça, which showed that a quarter of cases under treatment failed to adhere adequately, either by ceasing the treatment or delaying its completion by over 3 weeks[43].

Misconceptions of paediatric TB, associated complex care-seeking itineraries and negative feelings of the diagnostic procedures may result in delays, low adherence and lost to follow-up. In order to overcome these challenges, promotion should be informed by the aforementioned findings. Messages should emphasise that TB is not exclusively an adults′ illness, that the transmission is airborne, that although associated with HIV, they are distinct diseases and that due to the possibility of confounding with other illnesses it should be ruled out at first suspicion. Finally, a significant effort should be made to inform people of the lengthy and complex nature of TB diagnosis and treatment in order to encourage compliance with the formal system, despite the community′s tendency to seek alternative care.

This study has some limitations. Firstly, due to the recruitment method applied, all participants from groups A and B (except one) and all caretakers of group C had sought care at the hospital at some point in order to treat their TB compatible symptoms. Thus, there was not enough representation of those who do not ever seek health care in the formal sector. Secondly, it is unclear, particularly for those in Manhiça, the extent to which the research centre′s interviewer inhibited participants from talking openly about their alternative healthcare seeking practices, given that the research centre is intimately related to the district hospital and is often perceived to be aligned with the same formal health-care system objectives. Although this does not explain the discrepancy between the results of the two settings, it must be taken into consideration while assessing all interpretations.

## Conclusion

This study has shown that the knowledge of TB among caretakers of small children from this area of southern Mozambique is low. Paediatric TB is seldom suspected and often understood as an outcome of impurity derived from the transgression of social and cultural norms.

Misconceptions of paediatric TB, associated complex care-seeking itineraries and negative feelings of the diagnostic procedures may result in delays in diagnosis, low adherence and lost to follow-up, which needs to be addressed by adequately framed health promotion approaches.
